# Olfactory effects of a hypervariable multicomponent pheromone in the red-legged salamander, *Plethodon shermani*

**DOI:** 10.1371/journal.pone.0174370

**Published:** 2017-03-30

**Authors:** Damien B. Wilburn, Kari A. Doty, Adam J. Chouinard, Sarah L. Eddy, Sarah K. Woodley, Lynne D. Houck, Richard C. Feldhoff

**Affiliations:** 1 Dept of Biochemistry and Molecular Biology, University of Louisville, Louisville, Kentucky, United States of America; 2 Dept of Genome Sciences, University of Washington, Seattle, Washington, United States of America; 3 Dept of Zoology, Oregon State University, Corvallis, Oregon, United States of America; 4 Dept of Biological Sciences, Duquesne University, Pittsburgh, Pennsylvania, United States of America; Universidad de Chile, CHILE

## Abstract

Chemical communication via chemosensory signaling is an essential process for promoting and modifying reproductive behavior in many species. During courtship in plethodontid salamanders, males deliver a mixture of non-volatile proteinaceous pheromones that activate chemosensory neurons in the vomeronasal epithelium (VNE) and increase female receptivity. One component of this mixture, Plethodontid Modulating Factor (PMF), is a hypervariable pheromone expressed as more than 30 unique isoforms that differ between individual males—likely driven by co-evolution with female receptors to promote gene duplication and positive selection of the PMF gene complex. Courtship trials with females receiving different PMF isoform mixtures had variable effects on female mating receptivity, with only the most complex mixtures increasing receptivity, such that we believe that sufficient isoform diversity allows males to improve their reproductive success with any female in the mating population. The aim of this study was to test the effects of isoform variability on VNE neuron activation using the agmatine uptake assay. All isoform mixtures activated a similar number of neurons (>200% over background) except for a single purified PMF isoform (+17%). These data further support the hypothesis that PMF isoforms act synergistically in order to regulate female receptivity, and different putative mechanisms are discussed.

## Introduction

Information exchange in animals is mediated through a range of sensory systems—visual, auditory, chemical, vibrational, etc.—yet the broad steps can be generally described through a basic model of communication [1]: an information source produces a message, the message is encoded in a signal, the signal is broadcast, a receiver acquires the signal, and the signal is decoded. Arguably, chemical signaling—the most ancient form of cellular communication—relies on a “simple” system of direct biochemical interactions between ligands from a sender binding to target receptors in a receiver [2]. Olfaction is one form of chemical communication in vertebrates, and signal transduction is mediated through G-protein coupled receptors (GPCR) from three divergent but highly duplicated gene families [3, 4]. Of the various types of semiochemicals that may stimulate these receptors, pheromones are of particular interest for their ability to elicit preprogrammed behavioral or neuroendocrine responses [5, 6]. Despite >50 years of research [6], only a limited number of specific pheromone-receptor pairs have been identified [7–10]. A different but related challenge in pheromone research remains multicomponent signals: pheromones are generally released from glands as complex chemical mixtures [9, 11, 12], and pheromone activity is often dependent on the combination of several components in specific ratios [13, 14]. While multicomponent signals have been most well characterized in invertebrate systems, there has been limited investigation into how complex pheromone mixtures may influence vertebrate behaviors, and how these pathways are neurophysiologically mediated.

As basal tetrapods, salamanders are an excellent non-mammalian model to study the evolution and function of pheromone signaling in vertebrates [15]. For the salamander family Plethodontidae, male salamanders deliver non-volatile proteinaceous courtship pheromones from a submandibular gland to females in order to regulate courtship behavior and mating receptivity [16–18]. Preceding the annual mating season, plasma androgen levels rise in male salamanders and likely induce hypertrophication of a submandibular mental gland solely dedicated to the production of courtship pheromones [19–21]. In our principal model, the red-legged salamander (*Plethodon shermani*), male salamanders privately deliver pheromones to a female during an elaborate courtship ritual by “slapping” his hypertrophied mental gland to her nares [22]. Pheromones diffuse into the female nasal cavity, activate neurons in the vomeronasal epithelium (VNE) that project to regions of the brain classically involved in pheromone response, which stimulates changes in mating behavior [23–25]. During staged courtship trials, application of whole pheromone extract (WE) to female salamanders decreased tail straddling walk time by ~20% [26]. One major component of the pheromone is Plethodontid Modulating Factor (PMF), an ~7 kDa protein related to the highly diverse three-finger protein (TFP) superfamily, which includes snake venom cytotoxins and neurotoxins, the amphibian limb regeneration factor Prod1, and mammalian complement system receptor CD59 [20, 27–29]. Through gene duplication and pervasive positive selection, PMF is maintained as a multi-isoform blend [30], with individual male salamanders expressing more than 30 unique PMF isoforms with only ~30% sequence identity [31, 32]. PMF isoform expression levels vary between males, with three isoforms (G, H, and I; together defined as PMF-GHI) almost always found in greater abundance than a mixture of >30 minor isoforms (referred to as PMF-EF; see Wilburn et al. [31, 33] for details). In contrast, female behavioral response to PMF was different depending on the isoform composition: a single abundant PMF isoform (PMF-G) elicited no measurable effect, a mixture of minor isoforms (PMF-EF) increased courtship time, but a complete mixture of isoforms (PMF-EFGHI) decreased courtship time similarly to both WE and PRF [33, 34]. From these data, we hypothesized that PMF isoforms are acting synergistically to increase female receptivity through an unknown neurophysiological pathway. Hence, we sought to determine how these ultimate behavioral effects might be mediated at the level of olfactory stimulation.

Our working model is that PMF signals to the central nervous system via vomeronasal type-2 receptors (V2Rs) expressed on neurons in the VNE. Salamanders possess a single nasal cavity with the main olfactory epithelium and VNE divided along the medial and lateral edges, respectively [35]. Because of their large extracellular domains, V2Rs are thought to the principal receptors for peptide or protein pheromones in the VNE [4]. Notably, RT-PCR and *in situ* hybridization revealed high expression of V2Rs in the *P*. *shermani* VNE [36]. In order to measure neuronal activation in this system, the amino acid derivative agmatine (AGB) can be used as a tracer that passes through non-specific cation channels during membrane depolarization [37–40]. Co-application of pheromone and AGB to female salamanders results in selective uptake of AGB into activated neurons, and following tissue fixation, sectioning, and immunohistochemical labeling, a permanent record of neuronal activation is obtained [24]. Previous studies suggested that PRF and PMF-EF activate different subsets of VNE neurons, yet independently only accounted for ~70% of the activated neurons observed when females were treated with WE [41]. In seeking to understand how PMF isoforms function synergistically in affecting courtship behavior, it remained to be addressed whether each PMF-responsive VNE neuron can respond to a single or multiple PMF isoforms. If each neuron responded to a single isoform, we would expect proportional activation of VNE neurons based on the number of isoforms applied, and conclude that the behavioral synergistic effects are coordinated further along in the central processing. Otherwise, if more or fewer neurons are activated in proportion to the number of isoforms applied, we may anticipate functional synergy or redundancy, respectively, at the level of VNE activation. Therefore, to better characterize the role of PMF isoform diversity in regulating female courtship behavior, the aim of this study was to test the efficacy of different PMF isoform mixtures on stimulating female VNE neurons using the AGB uptake assay.

## Results

**Fig 1:**
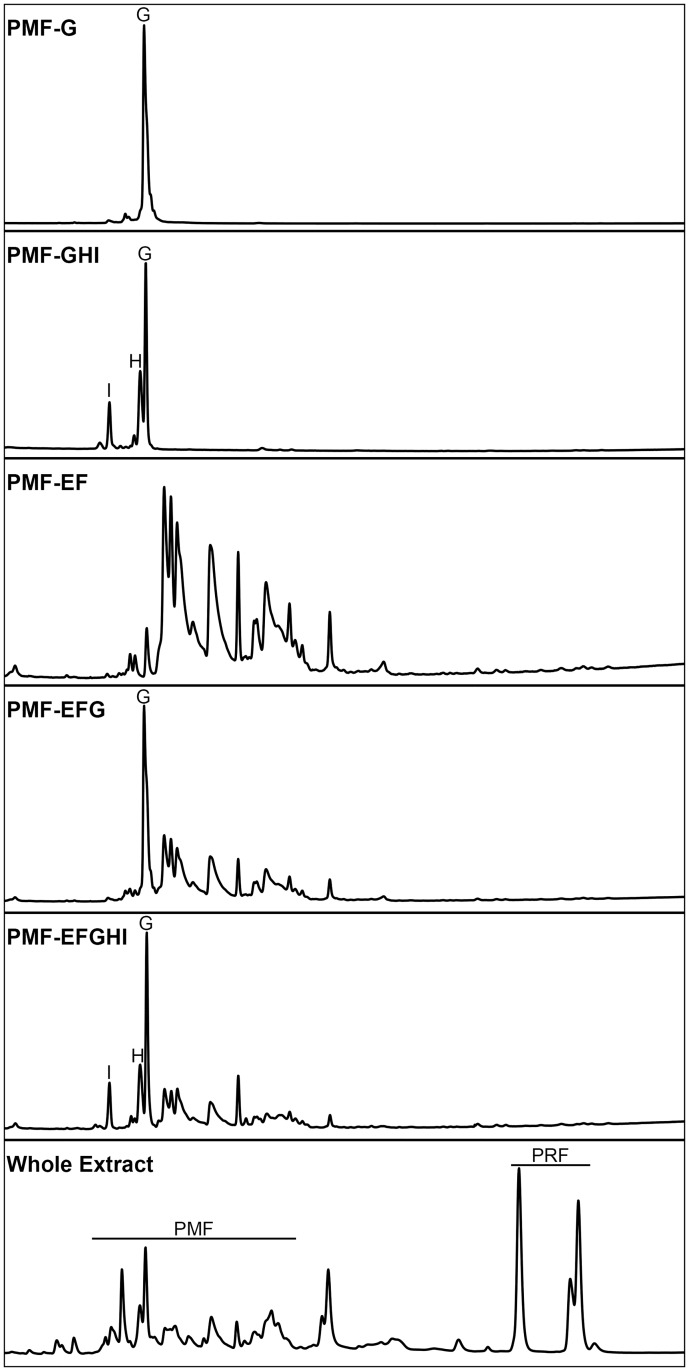
Pheromone isoform diversity. Representative reverse phase chromatograms of each tested pheromone treatment using a 0–70% acetonitrile gradient with 1% acetonitrile/min (25–60 min shown, adapted from Wilburn et al. [31]). Major PMF isoforms are denoted, as well as PMF and PRF in the whole pheromone extract.

**Fig 2:**
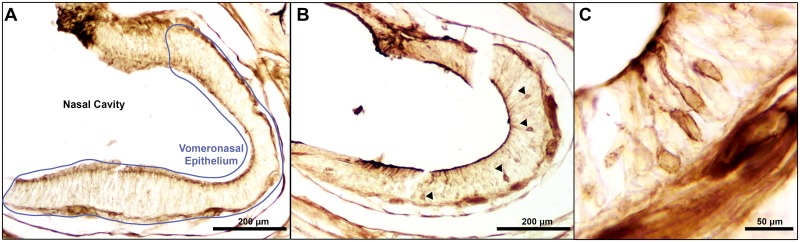
AGB immunohistochemistry. Transverse cross-sections of the olfactory chamber comparing the vomeronasal epithelium from females treated with 0.5X PBS (A) and WE (B), with arrows pointing to the cell bodies of immunoreactive neurons. (C) Higher magnification of four AGB neurons in close proximity. For greater detail on the architecture of the plethodontid olfactory system, see Wirsig-Wiechmann et al. [24].

**Fig 3:**
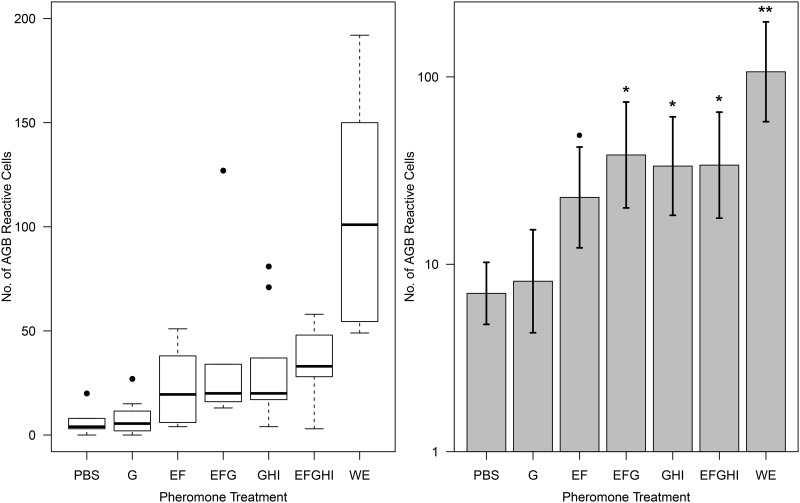
Summary of AGB neuron counts. (A) Box-and-whisker plots comparing the 7 pheromone treatments. The box defines the 25^th^ and 75^th^ percentiles, the median as the dark line, and whiskers as the minimum and maximum. Outliers are reported as values outside the median ± 1.5 * the interquartile range (box width). (B) Bar graph of the number of AGB reactive neurons per treatment (mean ± SE), with statistical significance relative to PBS denoted (●, p < 0.1; *, p < 0.05, **, p < 0.01)

Pheromone mixtures were prepared containing different degrees of PMF isoform diversity, with pheromone whole extract (WE) and 0.5X PBS used as a positive and negative controls, respectively (Fig 1). Prior to full immunohistochemical (IHC) processing, we optimized methods from the original Wirsig-Wiechmann et al. [24] protocol to reduce background staining and enhance resolution (Fig 2). Pheromone treatment significantly affected the number of AGB reactive neurons, determined by likelihood ratio test (χ^2^(6) = 38.5, p = 8.8 x 10^−7^). Comparison of effects are summarized in Table 1. As expected, WE activated the most neurons on average of any treatment ($\bar{x}=106.5$), and 0.5X PBS the fewest ($\bar{x}=7.0$). Three of the five PMF treatments (PMF-EFG, PMF-GHI, PMF-EFGHI) significantly activated more neurons relative to saline (Table 1, Fig 3), and all to a similar degree ($\bar{x}=38.3,\;33.4,\;33.8$ neurons, respectively). While PMF-EF was not significant at p < 0.05 ($\bar{x}=22.7,\;p=0.057$), the observed effect relative to 0.5X PBS (+225%) was nearly twice the effect observed in Wirsig-Wiechmann et al. [41] (+124%). Hence, this marginal p-value is likely a result of reduced power from the large number of treatment groups and correction for multiple comparisons. Except for PMF-G, there was no statistical difference between any of the PMF isoform mixtures.

**Table 1:** Summary of pheromone effects on AGB-immunoreactive cells.

	0.5X PBS	PMG-G	PMF-GHI	PMF-EF	PMF-EFG	PMF-EFGHI	WE
0.5X PBS	7.0	+16%	**+378%**	+225%	**+448%**	**+383%**	**+1421%**
PMF-G	0.81	8.1	**+312%**	+180%	**+372%**	**+316%**	+**1211%**
PMF-GHI	**0.0097**	**0.030**	33.4	-32%	+15%	+1%	+218%
PMF-EF	0.057	0.10	0.53	22.8	+68%	+49%	**+368%**
PMF-EFG	**0.0090**	**0.017**	0.83	0.42	38.3	-12%	+178%
PMF-EFGHI	**0.0016**	**0.029**	0.99	0.54	0.85	33.8	+215%
WE	**9.0 x 10**^**−6**^	**4.8 x 10**^**−5**^	0.059	**0.013**	0.12	0.078	106.5

Mean number of activated neurons per treatment are along the diagonal, the upper block listing the effect size as a percentage of column vs row, and the lower block reporting the p-values of differential labeling from 2-tailed Z-tests. Significant effects at p < 0.05 are denoted in bold.

## Discussion

In the current study, we evaluated the response of female vomeronasal neurons to different isoform combinations of PMF, a hypervariable salamander courtship pheromone. Behavioral studies of courtship indicate that PMF can either increase or decrease female receptivity depending on the isoform composition [33, 34]. The principal goal of this study was to determine if these different behaviors map proportionally onto VNE neuron activation. When the different mixtures of PMF isoforms were tested in the current study, all but the single isoform (PMF-G) elicited a similar response >200% over background. The non-significant +17% increase in neuronal activation from PMF-G was consistent with courtship trials where there was no detectable response over vehicle [33]. However, the mixture of the three highly abundant isoforms, PMF-GHI, elicited a robust response compared to G alone, suggesting that (1) PMF-H and PMF-I are independently producing large effects, or (2) PMF isoforms act synergistically to stimulate females. While we cannot definitively exclude the hypothesis that PMF-HI has a large independent effect, this seems unlikely for several reasons. First, PMF-G is consistently more abundant than PMF-H or PMF-I in the pheromone extract [31]. Second, the three-dimensional structure of PMF-G was recently solved by NMR, with homology modeling suggesting that PMF-H and PMF-I are structurally very similar to PMF-G such that they may bind similar receptors [42]. Third, the only solution missing PMF-G (PMF-EF) activated the fewer neurons compared to other PMF mixtures, and while not statistically significant, it is interesting that the same mixture plus PMF-G (PMF-EFG) activated approximately twice as many neurons. Consequently, these data further support the hypothesis that synergistic interactions between different PMF isoforms are required in order to enhance female receptivity, but not in a linear relationship between VNE neuron activation and reduction in courtship time.

One quality of PMF that seems common to multiple vertebrate protein pheromones is evolutionary histories with pervasive gene duplication and neofunctionalization. In mice, both the major urinary proteins (MUPs) and exocrine gland-secreting peptide (ESP) families are highly polymorphic, with isoforms variably expressed both within and between inbred strains [11]. Many of these isoforms may have unique functions: specific MUPs are involved in regulating female receptivity and promoting male aggressive behavior [43, 44]; and the male-specific ESP1, but not other ESPs, increased female receptivity [9]. In contrast to these mouse systems, where the pheromones are dispersed into the environment as part of general bodily fluids, the mental gland of plethodontid salamanders is only used to privately deliver signals to a single female during courtship [45]. Based on the timing of mental gland development and its use during tail-straddling walk, these pheromones presumably serve no functions beyond regulating behaviors that may impact reproductive success [23, 46]. Therefore, unlike the mouse pheromones with disparate functions, the many isoforms of both PMF and PRF may be acting on overlapping biological pathways, which fits within the “redundant-signal” hypothesis [47, 48].

However, the precise mechanism by which these PMF molecules are interacting to activate VNE neurons and affect female behavior remains unclear. All available evidence supports the hypothesis that PMF binds to V2Rs in the female VNE [36, 41]. In mice, pheromone binding to V2Rs induces the IP_3_ signaling cascade that eventually leads to opening of the transient cation channel TRPC2, allowing an influx of Ca^2+^, membrane depolarization, and signal transduction to the central nervous system [49, 50]. The proposed mechanism of AGB uptake during neuronal depolarization is via open TRPC2 channels following pheromone binding. Critically, TRPC2 is highly abundant in the *P*. *shermani* VNO [36]. Recent work has shown AGB can function as both a neurotransmitter and a tracer molecule [51], such that it is possible we are partially observing an synergistic response between pheromone and AGB in the female VNE. However, previous work by Laberge et al. [25] showed that PMF activates the vomeronasal amygdala (downstream of the VNE in the accessory olfactory system), as well as the preoptic area and ventromedial hypothalamus (which are often involved in reproductive behavior). In addition to a full suite of IHC controls [52], we believe there is little to no interaction between pheromone and AGB in the female VNE. Both NMR and analytical ultracentrifugation studies demonstrated that PMF is monomeric, both as a single isoform and a complex mixture [42]. While some TFPs can form dimers [29], it is likely that the high negative charge density of PMF precludes such interactions [42]. One possible explanation may be that different PMF isoforms form a multimeric complex with a single receptor molecule. Compared to other members of the three-finger protein superfamily, PMF-G has a novel protein topology and three-dimensional structure that permits greater backbone flexibility in one of the three fingers, which is also the most variable and rapidly evolving segment in the PMF gene complex [42]. With less structural variation in the rest of the molecule, this may allow for any number of different isoforms to dock with target receptors through the conserved regions, but activation may be dependent on proper interactions from the variable third fingers of the necessary isoforms in the proper orientation. These potential kinetic constraints may help explain the selection pressure for the large number of PMF isoforms (>30 expressed between different male *P*. *shermani*) and their large abundance in the mental gland (~5:1 stoichiometry compared to PRF, and ~50% of total pheromone in whole extract) [31, 32].

However, one previously unexplored hypothesis is that some isoforms of PMF may be functioning as signature mixtures rather than pheromones. The classic Karlson and Luscher [6] definition of a pheromone is a molecule that elicits a pre-programmed behavioral and/or neuroendocrine effect. In contrast, signature mixtures, proposed by Wyatt [5], are variable sets of cues that provide information on identity of specific individuals in a population, but elicit no innate, pre-programmed response in receiving individuals. What makes these terms somewhat confounding is that, under the proper conditions, signature mixtures can function similarly to pheromones. For example, the mouse pheromone darcin—a MUP isoform delivered alongside many other proteins and volatile odorants in mouse urine—provokes learning of male odor profiles in virgin females such that, upon subsequent exposure to the same odor profile minus darcin, female receptivity increases. In this system, females are now conditioned to respond to particular markers, but without initial co-exposure with darcin, there is no learning of these individual cues [44, 53]. In *P*. *shermani*, male salamanders express unique, individual PMF profiles visible by high performance liquid chromatography (HPLC). Although the exact composition of these mixtures is hard to biochemically dissect, PMF-G, H, and I seem to be universally expressed at proportionally high levels [32]. Comparison of PMF cDNA sequences revealed that while there exists more than 99 unique putative isoforms in a single population, sequences were more accurately clustered into 13 common archetypes with only 1–2 SNPs varying between the sequences within each cluster [31]. Each of these PMF isoform clusters may be performing unique roles, either by targeting different classes of receptors or functioning as part of a signature mixture to convey identity. Because learning is an active component of signature mixture response [5], and *P*. *shermani* are difficult to breed under laboratory conditions (L.D. Houck, personal communication), it is a challenge to test these variable pheromone components on virgin females. However, female plethodontid salamanders can distinguish between the odors of individual conspecifics [54]. Additionally, learning of predator cues has been tested in multiple amphibian species [55–57], and in mice, many of these heterospecific signals are mediated through specific vomeronasal receptors (both V1Rs and V2Rs) [58]. Common isoforms such as PMF-G may be acting as more traditional pheromones, while other variable PMFs serve more as individual cues in signature mixtures. This hypothesis is complementary to the longer standing view that PMF has been subject to extensive gene duplication and pervasive positive selection in order to expand the functional breadth of PMF as a “pheromone” [30, 31]. Testing this hypothesis will be difficult and likely require repeated measurements on initially virgin female salamanders with careful manipulation of pheromone composition using recombinant proteins.

In summary, PMF is a hypervariable vertebrate courtship pheromone that differentially regulates female courtship behavior at least in part through variability in isoform composition. For the first time, we demonstrated that a single isoform of PMF (G) was unable to significantly activate neurons in the female VNE; however, any mixture of PMF containing 3 or more isoforms elicited a similar response of ~350% activation over vehicle. The exact receptors and mechanisms mediating this response are still unclear. Multiple hypotheses to explain this phenomenon were presented, including PMF isoforms forming multimeric complexes with V2Rs, VNE neurons expressing multiple V2Rs, and/or learning in female salamanders as part of signature mixtures. Future studies will seek to further characterize the molecular architecture of the *P*. *shermani* VNE, identifying the specific PMF receptors, and elucidating the binding mechanics in order to better understand the evolutionary forces that have driven exacerbated gene duplication and positive sexual selection on PMF over the past 100 million years.

## Materials and methods

### Animals collection

*P*. *shermani* salamanders were collected from a single site in Macon Co., North Carolina, USA (35°10’48” N, 83°33’38” W) during their annual breeding season, and housed at Highlands Biological Station for duration of the experiment. Adult salamanders were identified and sexed based on a well-developed mental gland in males and large ova in females. Animals were individually housed at 15–18°C and ~70% humidity in clean plastic boxes (17 x 9 x 13 cm) lined with a damp paper towel, and a second damp crumpled paper towel for cover. Once per week, salamanders were transferred to clean boxes with new substrate and fed 2 waxworms (*Galleria mellonella*). All animals were collected under permits obtained from the North Carolina Wildlife Resources Commission, and all animal methods were approved by Oregon State University ACUP #4053 to L.D. Houck.

### Preparation of male pheromones

Whole pheromone extract (WE) was collected from male salamanders following the methods of Rollmann et al. [23]. Briefly, male salamanders were anesthetized in a mixture of 7% ether/water for ~7 minutes. As *P*. *shermani* are lungless salamanders, there are few options for anesthetics compared to mammals, and the use of diethyl ether has been part our approved IACUC protocols for more than 20 years. In unpublished work, we evaluated the use of both diethyl ether and tricaine methanesulfonate (MS-222) on *P*. *shermani*, and we found that when using ether: time to anesthetization was more consistent between animals; there was lower risk of bleeding during mental gland removal; and more pheromone was extracted. The mental gland was then surgically removed from the dermis using iridectomy scissors, and incubated in acetylcholine chloride (0.8 mM in amphibian Ringer’s solution) for 60 minutes to induce pheromone secretion. Mental glands were removed by two rounds of centrifugation at 10,000 *x g* for 10 min, and pheromone extracts stored at -80°C until further processing at the University of Louisville. WE was prepared by ultrafiltration and standardized at 2.0 mg/mL in 0.5X phosphate buffered saline (PBS). PMF-EF and PMF-EFGHI were prepared based on the methods of Wilburn et al. [31] using HPLC. Briefly, WE was subjected to strong anion exchange HPLC in order to collect the PMF-EFGHI fraction. PMF-EF and PMF-GHI were resolved by a second round of strong anion exchange HPLC with a shallower elution gradient. PMF-GHI was then purified to >99% purity by one round of reverse phase HPLC such that the solution only contained the three most abundant PMF isoforms. PMF-EFG was prepared by mixing natural PMF-EF with recombinant PMF-G in a 7:2 ratio (which approximates natural levels, based on integration of peak areas observed by strong anion exchange HPLC). Highly purified recombinant PMF-G was prepared based on the methods of Wilburn et al. [42] using the methylotrophic yeast *Pichia pastoris*. All biochemical and structural studies have confirmed that recombinant PMF-G has an identical sequence and 3D structure to natural PMF-G, and is thus suitable for bioassays. All PMF solutions were standardized using ultrafiltration to 0.5 mg/mL in 0.5X PBS.

### AGB uptake assay and immunohistochemistry

The AGB uptake assay was performed based on the methods from Wirsig-Wiechmann et al. [24]. Pheromone solutions were mixed 1:1 with 6 mM AGB (Sigma Aldrich, St. Louis, MO) in 0.5X PBS. A total of 70 adult gravid female salamanders were placed in new, unused Tupperware sandwich boxes lined with a single damp paper towel and allowed to acclimate for 30 min. Each salamander received one of 7 different treatments (n = 10 per treatment): 0.5X PBS (negative control/vehicle), PMF-G, PMF-GHI, PMF-EF, PMF-EFG, PMF-EFGHI, or WE (positive control). Two microliters of pheromone/AGB was applied to the female’s nares every 2 minutes for a total 40 min (20 applications), followed by 3 rinses with 5 μL 0.5X PBS over ~5 minutes. Females were then rapidly decapitated, the lower jaw removed, and heads incubated overnight in 10mL 4% paraformaldehyde/2.5% glutaraldehyde (in 150mM sodium chloride/100mM sodium phosphate, pH 7.4). Heads were then decalcified using DeCal (Decal Corporation, Congers, NY) for 3 days, cryoprotected in 30% sucrose for 2 days, and embedded using Optimal Cutting Temperature (OCT) media (Sakura-Finetek, Torrance, CA). A total of 14 blocks were prepared with 5 heads each, and stored at -80°C prior to cryosectioning. Heads were sectioned coronally at a thickness of 20 μm, and thaw mounted onto superfrost plus slides pre-coated with polylysine. Sections were collected in four sets such that each section in a set was separated by 80 μm. However, due to a cryostat malfunction, a large percentage of sections were lost for three blocks, and were excluded from the analysis (effective n = 55). Slides were stored at -80°C prior to immunohistochemistry. One slide set was used to optimize IHC conditions from the original Wirsig-Wiechmann et al. [24] protocol to reduce background staining (adjustments were principally in the concentration of detergents used in pre-incubation and washing steps): after equilibration to room temperature, slides were washed five times for 5 min each in 1X PBS, preincubated in 1% normal goat serum/1X PBS/0.2% Triton X-100/0.5% Tween-20/0.02% azide for 30 min, and incubated in rabbit anti-AGB (EMD-Millipore, Billerica, MA) diluted 1:2000 in 1% normal goat serum/1X PBS/0.1% Triton X-100/0.05% Tween-20/0.02% azide for three days at room temperature. Slides were then washed five times for 5 min each in 1X PBS/0.05% Tween-20 (PBST), incubated for 30 minutes in biotinylated goat anti-rabbit IgG (Thermo-Pierce, Rockford, IL) diluted 1:500 in PBST, washed five times for 5 min each with PBST, incubated in 0.5X ultra-sensitive ABC peroxidase staining reagent (Thermo-Pierce) in PBST for 30 minutes, washed three times for 5 min each with PBST, twice with 1X PBS for 5 min each, and developed with metal enhanced DAB (Thermo-Pierce) for 5 min before serial dehydration and coverslipped with Permount (Fisher Scientific, Pittsburgh, PA).

### Histological and statistical analyses

Slides were visualized and imaged using an Olympus microscope with an attached 9 megapixel digital camera. AGB reactive neurons were counted in VNE tissue for all sections from both left and right nasal cavities, and the investigator was blind to the treatments. Count data was analyzed using generalized linear models with negative binomial distributions using the R function glm.nb in the package *MASS*. The overdispersion parameter τ was estimated to be 1.716. The effect of pheromone treatment was evaluated by likelihood ratio test against an intercept-only model, with individual effects of the seven solutions/levels examined *post-hoc* by z-test with corrected standard errors.

## Supporting information

S1 File Counts of AGB reactive neurons. (CSV)
